# Author Correction: Epitaxial growth of an atom-thin layer on a LiNi_0.5_Mn_1.5_O_4_ cathode for stable Li-ion battery cycling

**DOI:** 10.1038/s41467-022-33219-7

**Published:** 2022-09-16

**Authors:** Xiaobo Zhu, Tobias U. Schülli, Xiaowei Yang, Tongen Lin, Yuxiang Hu, Ningyan Cheng, Hiroki Fujii, Kiyoshi Ozawa, Bruce Cowie, Qinfen Gu, Si Zhou, Zhenxiang Cheng, Yi Du, Lianzhou Wang

**Affiliations:** 1 Nanomaterials Centre, School of Chemical Engineering, and Australian Institute of Bioengineering and Nanotechnology, The University of Queensland, Brisbane, QLD 4072 Australia; 2 College of Materials Science and Engineering, Changsha University of Science and Technology, Changsha, 410114 China; 3 ESRF—The European Synchrotron, 38000 Grenoble, France; 4 Key Laboratory of Materials Modification by Laser, Ion and Electron Beams (Dalian University of Technology), Ministry of Education, Dalian, 116024 China; 5 Australian Institute for Innovative Materials (AIIM), University of Wollongong, Squires Way, North Wollongong, NSW 2500 Australia; 6 National Institute for Materials Science, 1-2-1 Sengen, Tsukuba-city, Ibaraki, 305-0047 Japan; 7 Australian Synchrotron, 800 Blackburn Road, Clayton, VIC 3168 Australia

**Keywords:** Inorganic chemistry, Nanoscale materials, Batteries, Energy storage

Correction to: *Nature Communications* 10.1038/s41467-022-28963-9, published online 23 March 2022

The original version of this article contained an error in Supplementary Fig. 23, in which the two XPS spectra of C*1s* were mistakenly duplicated. The original article has been corrected.

## Supplementary information

Updated Supplementary Information

