# Supplementary material for: Epitaxial growth of an atom-thin layer on a LiNi0.5Mn1.5O4 cathode for stable Li-ion battery cycling
Source: Nat Commun. 2022 Mar 23;13:1565. doi: 10.1038/s41467-022-28963-9 (PMC8943144; doi:10.1038/s41467-022-28963-9)
Supplement: Supplementary file 1 — Supplementary Information [file 41467_2022_28963_MOESM1_ESM.pdf]

Supplementary Information

Title

Epitaxial growth of an atom-thin layer on a $LiNi_{0.5}Mn_{1.5}O_4$ cathode for stable Li-ion battery cycling

Authors

Xiaobo Zhu[1, 2], Tobias U. Schülli[1, 3]*, Xiaowei Yang,[4] Tongen Lin,[1] Yuxiang Hu,[1] Ningyan Cheng,[5] Hiroki Fujii,[6] Kiyoshi Ozawa,[6] Bruce Cowie,[7] Qinfen Gu,[7] Si Zhou,[4, 5] Zhenxiang Cheng,[5] Yi Du,[5] Lianzhou Wang[1]*

Affiliations

[1]Nanomaterials Centre, School of Chemical Engineering, and Australian Institute of Bioengineering and Nanotechnology, The University of Queensland, QLD 4072 Australia

[2]College of Materials Science and Engineering, Changsha University of Science and Technology, Changsha 410114, China

[3]ESRF – The European Synchrotron 38000 Grenoble, France

[4]Key Laboratory of Materials Modification by Laser, Ion and Electron Beams (Dalian University of Technology), Ministry of Education, Dalian 116024, China

[5]Australian Institute for Innovative Materials (AIIM), University of Wollongong, Squires Way, North Wollongong NSW 2500

[6]National Institute for Materials Science, 1-2-1 Sengen, Tsukuba-city, Ibaraki, 305-0047, Japan

[7]Australian Synchrotron, 800 Blackburn Road, Clayton, VIC, 3168 Australia

Inventory of Supplementary Information

Supplementary Note 1
Supplementary Figures 1 to 25
Supplementary Tables 1 and 2
Supplementary References

Supplementary Note 1

Quantification of La uptake ion the LNMO surfaces:

In order to evaluate the amount of La that is absent from the formed secondary $LaTMO_3$ phase, two independent quantification methods based on XRD have been applied.

I) With the integrated intensity of a Bragg peak being proportional to the amount of scattering volume a simple plot of *e.g.* the integrated intensity of the cubic perovskite $LaTMO_3$ $Pm\bar{3}m$ (110) reflection should have a linear evolution as a function of La content X_{La}in the precursor mix, in case all La can be found in the perovskite phase and none in the LNMO Spinel phase. Based on this assumption, and in case this formation of extra phases only sets in above a certain critical threshold X_{La_c} a linear interpolation of the observed integrated intensities allows to precisely determine this threshold.

If based on the integrated intensity of the $Pm\bar{3}m$ (110) reflection one also has to take into account an eventual overlap with $LaTMO_3$ crystallizing in the rhombohedral $R\bar{3}c$ phase leading to the formation of the (104) and $(2\bar{1}0)$ double peak. Considering experimental conditions at an X-ray energy of 18 keV, we obtain for the cubic phase the structure factor of $F_{110(c)}$=61.4 and for the $R\bar{3}c$ phase $F_{104(t)} = 373.2$ and $F_{2\bar{1}0(t)} = 365.4$. This yields $F_{104(t)}$+$F_{2\bar{1}0(t)} \approx 12 * F_{110(c)}$. Taking into account the about 6 times bigger unit cell volume and the peak multiplicities (12 for the $Pm\bar{3}m$ phase and 6 for the $R\bar{3}c$ phase) it turns out that the integrated intensity in the powder spectrum from these peaks all together remains proportional to the volume of these extra $LaTMO_3$ phases formed. Seen the similarity of the two structures this is not surprising.

II) The second way of quantification is based on a comparison between the integrated intensities of the above extra phase peak(s) with *e.g* the neighboring (113) peak of the LNMO spinel phase. With the crystallographic knowledge of the volumetric density of TM and La atoms in both structures, this allows a precise determination of the relative volume occupied by both structures. Comparing this to the fraction X_{La} present in the precursors an eventual discrepancy between the La present in the perovskite phase(s) can also evaluate X_{La_c} which is consumed in the strained epitaxial surface layer ontop of the LNMO (111) facets. As the intention is to compare atomic fractions of La/TM, it is practical to determine the structure factor F_{hkl} per unit volume F_{hkl}^{Vol} in order to derive an expected Bragg peak intensity ratio between peaks of LNMO and $LaTMO_3$ as a function of their relative volumes. With the crystallographic information these relative volumes can then be easily translated in relative atomic fractions of La and TM atoms. With the cubic lattice parameters $a_{LNMO} = 0.817\ nm$ and $a_{LaTMO_3} = 0.389\ nm$ one obtains for an X-ray energy of 18 keV

$$F_{113_{LNMO}}^{Vol} = \frac{155.96}{(0.817\ nm)^3} = 0.000286\ nm^{-3} \qquad \text{Eq. 1}$$

and $$F_{110_{LaTMO_3}}^{Vol} = \frac{61.4}{(0.389\ nm)^3} = 0.00104\ nm^{-3} \qquad \text{Eq. 2}$$

The intensity ratios can then be written as

$$\frac{I^{110}_{LaTMO_3}}{I^{113}_{LMNO}} = \frac{\left|F^{Vol}_{110_{LaTMO_3}}\right|^2 * m_{110}}{\left|F^{Vol}_{113_{LMNO}}\right|^2 * m_{113}} \frac{V_{LaTMO_3}}{V_{LMNO}} \frac{LP_{110}}{LP_{113}}, \qquad \text{Eq. 3}$$

With m_{hkl} representing the peak multiplicity and LP_{hkl} the Lorentz- and polarization correction resulting from the scattering geometry. In our case it is clear that X_{La} will impact the Volume ratio. Due to the structure of $LaTMO_3$ and the definition of X_{La} with respect to the amount of TM atoms in the precursor mix, one can express this volume ratio as a function of X_{La}:

$$\frac{V_{LaTMO_3}}{V_{LMNO}} = \frac{X_{La} * \rho^{La}_{LaTMO_3}}{(1-2*X_{La}) * \rho^{TM}_{LMNO}} \approx \frac{X_{La} * \rho^{La}_{LaTMO_3}}{\rho^{TM}_{LMNO}}, \qquad \text{Eq. 4}$$

with $\rho^{\frac{TM}{La}}_{phase}$ being the volumetric densities of La in $LaTMO_3$ and TM in LNMO. In the denominator X_{La} is taken into account twice, as one TM atom per La atom is transferred to the $LaTMO_3$ counting only the TM atoms in the LNMO and only the La atoms in the $LaTMO_3$. For small amounts of X_{La} however the approximation in the last step can be used. This will well represent the expected La/TM ratio in case all La can be found in the $LaTMO_3$. Eqs. 2 and 3 combine to

$$X_{La} = \frac{I^{110}_{LaTMO_3}}{I^{113}_{LMNO}} \frac{V_{LMNO}}{V_{LaTMO_3}} \frac{\left|F^{Vol}_{113_{LMNO}}\right|^2 * m_{113}}{\left|F^{Vol}_{110_{LaTMO_3}}\right|^2 * m_{110}} \frac{LP_{113}}{LP_{110}} \qquad \text{Eq. 5}$$

The close neighborhood in angular space of the chosen peaks makes this quantification robust and allows us to neglect effects of thermal vibration (Debye-Waller factor) on the intensities of the Bragg peaks. Eq. 4 allows thus a direct extraction of the fraction of X_{La} in the $LaTMO_3$ phase. The difference between this fraction and the amount of La originally present in the precursor mix corresponds to the amount of La consumed by the epitaxial layer on-top of the LNMO crystals. It is equivalent to the amount of La X_{La_c} corresponding to the nucleation threshold mentioned above.

Energetic considerations of surface coverage by a strained layer and successive island formation.

Like in hetero-epitaxial growth, a vastly studied process, it is a question of surface energy vs. elastic energy that determines how many strained layers grow successively before onset of island growth. Islands increase the effective surface, but their relaxation reduces the elastic energy in the system. The thickness of the deposited layer depends on the mismatch of the two lattices, the miscibility of the constituents and the growth temperature, responsible for miscibility (reducing island formation) and surface diffusion (favoring island formation). These processes have been exhaustively studied for systems like Ge on Si or InAs on GaAs that have mismatches of 4.2 and 7 % respectively with a variable degree of miscibility at typical growth temperatures between 500 and 700 °C *(1-3)*. In our case we observed immiscibility of La in LNMO. Together with the high synthesis temperatures, this is kinetically favorable for island growth if the latter is of energetic advantage.

The driving force of island formation is a reduction of elastic energy. The minimum size of an island to be formed depends on the ratio of increase of free surface and the reduction of elastic

energy due to the removal of a strained atomic layer at the surface. The islands in this work grow fully relaxed as observed by XRD. Let us first consider the elastic energy per surface area of a biaxially strained thin film by introducing the biaxial elastic modulus $E^{`}$ in the form

$$E^{`} = \frac{E}{1-\nu} \qquad \text{Eq. 6}$$

With E being the Youngs Modulus and ν the Poisson ratio of the considered material. The elastic energy per surface area W_{eS} of a biaxially expanded sheet of thickness t and strain ε can then be calculated assuming linear elasticity *(4)*:

$$W_{eS} = \varepsilon^2 E^{`} t \qquad \text{Eq. 7}$$

Typical values taken from literature for $LaMnO_3$ vary a lot depending on the analysis method. The open source software ELATE *(5)*: yields $E = 212\ GPa$ and $\nu = 0.28$, resulting in $E^{`} = 294\ GPa$. When considering atomic layer coverages of $t \approx 0.22\ nm$ (the crystallographic spacing between 2 La containing planes is 0.22 nm), the elastic energy in a 5 % distorted layer is thus $W_{eS}\ (t = 0.22\ nm)$ = 0.16 J m^{-2}.

For comparison, the binding energy (E_b) between La-Mn-O (La-Ni-O) monolayer and LNMO substrate is -8.64 eV (-6.58 eV) for per formula given by DFT calculations, which is defined as:

$$E_b = E_{total} - E_{La\text{-}TM\text{-}O} - E_{substrate} \qquad \text{Eq. 8}$$

where E_{total}, $E_{La\text{-}TM\text{-}O}$ and $E_{substrate}$ are the energies of the La-TM-O/LNMO hybrid system, La-TM-O monolayer, and LNMO substrate, respectively. The E_b is equivalent to 0.29 J m^{-2} (0.22 J m^{-2}). It is thus the interfacial binding energy that explains wetting and the thermodynamic stability of the wetting layer.

Once the first atomic layer is deposited, the successive layers do not impact the surface energy anymore but add to the elastic energy budget by increasing t of the wetting layer. This further increase in elastic energy is proportional to the deposited volume. If surface diffusion is activated due to high enough temperatures, an alternative is island formation, increasing the effective surface and hence the surface energy but decreasing the elastic energy as the atomic lattice, even for coherent interfaces, can at least partially relax. This growth mode is referred to as Stranski Krastanow growth mode and very common in lattice mismatched heteroepitaxy. In our case, full relaxation through interface dislocations is observed. This makes an estimation of the interest of island formation particularly easy. A rough estimation is done by taking *e.g.* the volume of a trigonal pyramid as a "cube-tip" shaped island as it is expected to grow on a (111) surface with (001) side walls (due to the low $LaMnO_3$ (001) surface energy of about 0.9 J/m^2 *(32)* of side length l: $V_l = \frac{\sqrt{2}l^3}{24}$. The increase in surface ΔS_L as compared to e.g a second deposited monolayer corresponds to three trigonal facets: $\Delta S_L = \frac{3}{4} l^2$. The critical size l_c for the smallest island to be energetically favorable can be determined by taking the difference ΔE between increase in surface energy and decrease in elastic energy:

$$\Delta E = E_S \frac{3}{4} l^2 - W_{eV} \frac{\sqrt{2}l^3}{24} \qquad \text{Eq. 9}$$

With E_S~0.9 J/m^2, as a the $LaMnO_3$ (001) surface energy and $W_{eV}=\varepsilon^2 E^`$=7.4*10^8 J/m^3. Figure S11 presents a plot of ΔE as a function of l. For $\Delta E > 0$ island formation increases the systems total energy, for ΔE<0 island formation leads to decrease in total energy. Here formation of an island with typical side length of 7.5 nm or more is energetically favored. For comparison, the island volume in this case would remove a structural monolayer of $LaTMO_3$ over an area of approximately 2000 nm^2 corresponding to an area of about a single facet of a 40 nm side length octahedron.

The observation of such epitaxial islands in general proves that the growth temperature allows for ample surface diffusion, even for bulk diffusion as is shown by the partial expulsion of La in case of a doping procedure. This surface diffusion guarantees that the monolayer covers the complete free surface of the LNMO crystals, supplying an essentially void-free epitaxial coating. Other deposition methods as ALD often require lower process temperatures to avoid interdiffusion, as the deposited material does not allow for an epitaxial growth under equilibrium conditions. This means that at higher temperatures Vollmer-Weber growth would lead to immediate island formation and complete de-wetting of the deposited layers, exposing the surface to be protected. But this absence of diffusion also prevents a self-organized and void free coating which in our case is favored by diffusion.

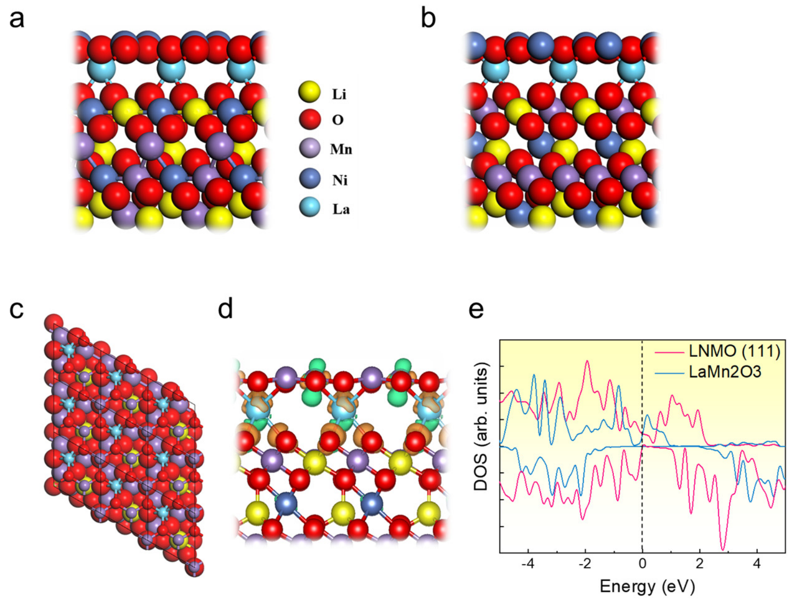

Supplementary Figure 1. DFT calculations for monolayer La-Ni-O and La-Mn-O on LNMO (111) substrate. Model structures of monolayer $LaNi_2O_3$ on LNMO (111) substrate by DFT calculations. The Ni dopants are either on the surface (a) or in the interior (b) of the substrate, respectively, with the latter system lower in energy by 1.60 eV than the former one. Model structures (top view) (c), differential charge density (side view) (d) and spin-polarized density of states (e) of monolayer $LaMn_2O_3$ on LNMO (111) substrate by DFT calculations.

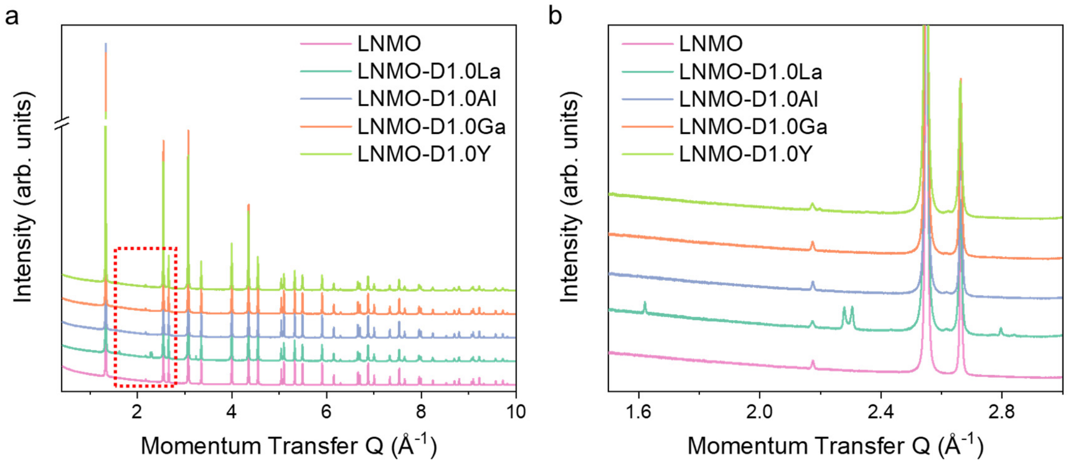

Supplementary Figure 2. Integration of different metals in LNMO with a doping method. (a) Synchrotron XRD patterns of LNMO products involving different metals by the doping way. LNMO-D1.0La, LNMO-D1.0Al, LNMO-D1.0Ga and LNMO-D1.0Y represent LNMO doped with 1.0 at% La, Al, Ga and Y, respectively. (b) Zoom of at the dashed red frame in (a) showing "typical" metal oxide Bragg peaks in order to give better visibility to eventual extra phases.

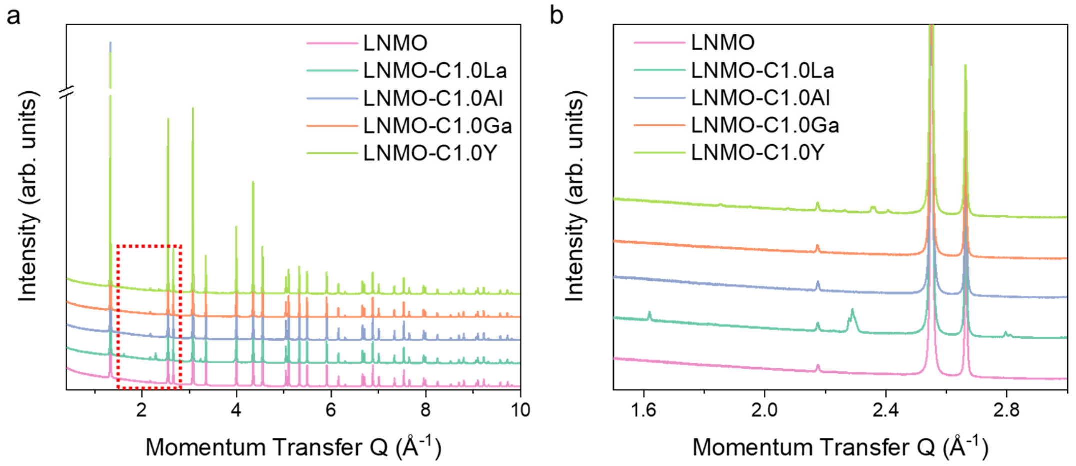

Supplementary Figure 3. Integration of different metals in LNMO with a coating process. (a) Synchrotron XRD patterns of LNMO products incoporting different metals by the coating method. LNMO-C1.0La, LNMO-C1.0Al, LNMO-C1.0Ga and LNMO-C1.0Y represent LNMO coated with 1.0 at% La, Al, Ga and Y, respectively. (b) Zoom of at the dashed red frame in (a) showing "typical" metal oxide Bragg peaks in order to give better visibility to eventual extra phases.

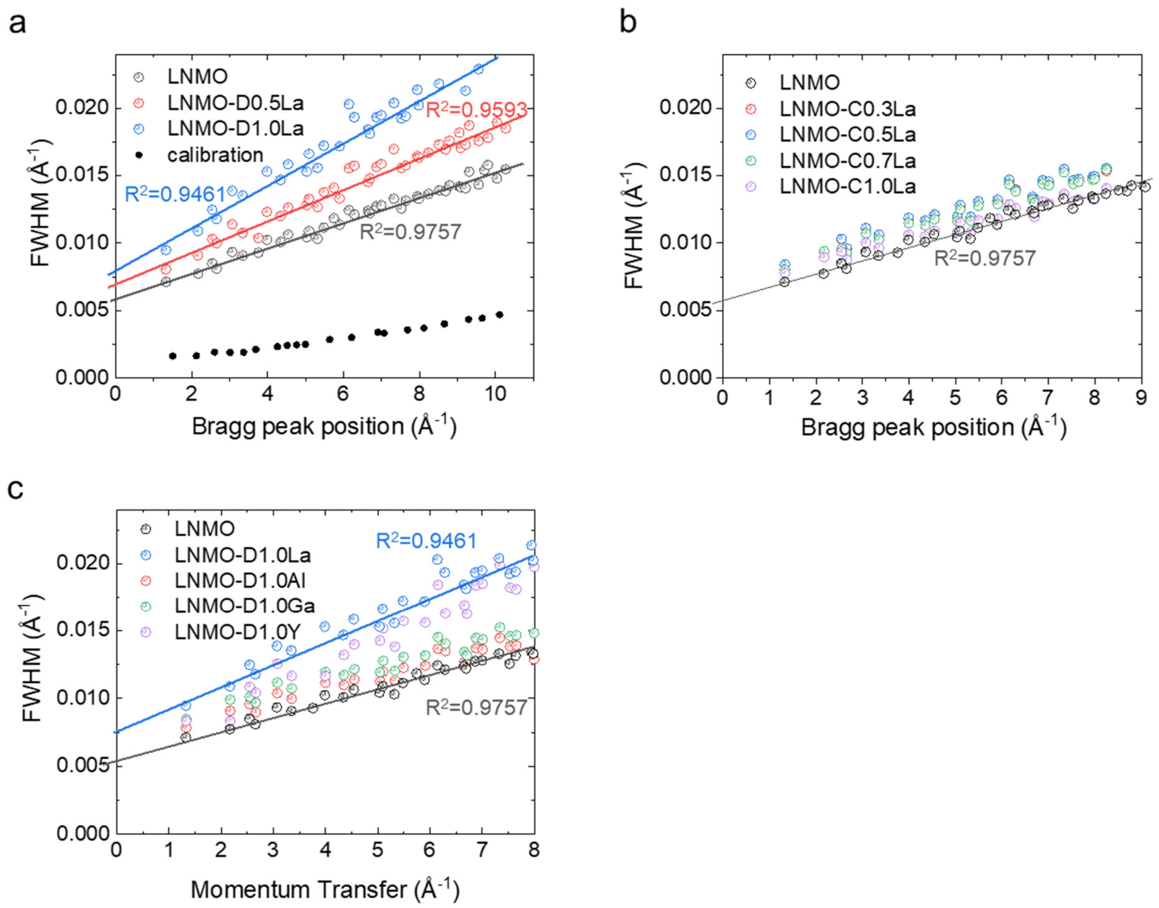

Supplementary Figure 4. Influence of different metals and methods on the LNMO crystallinity. XRD profile analyses showing the influence of the La doping (a), the La coating (b) and doping by different metals (c) on the crystal quality of resultant LNMOs. Plotted are the FWHM of Bragg peaks in reciprocal space as a function of their position. The fitting errors (R^2) are 0.9757, 0.9593 and 0.9461 for LNMO, LNMO-D0.5La and LNMO-D1.0La.

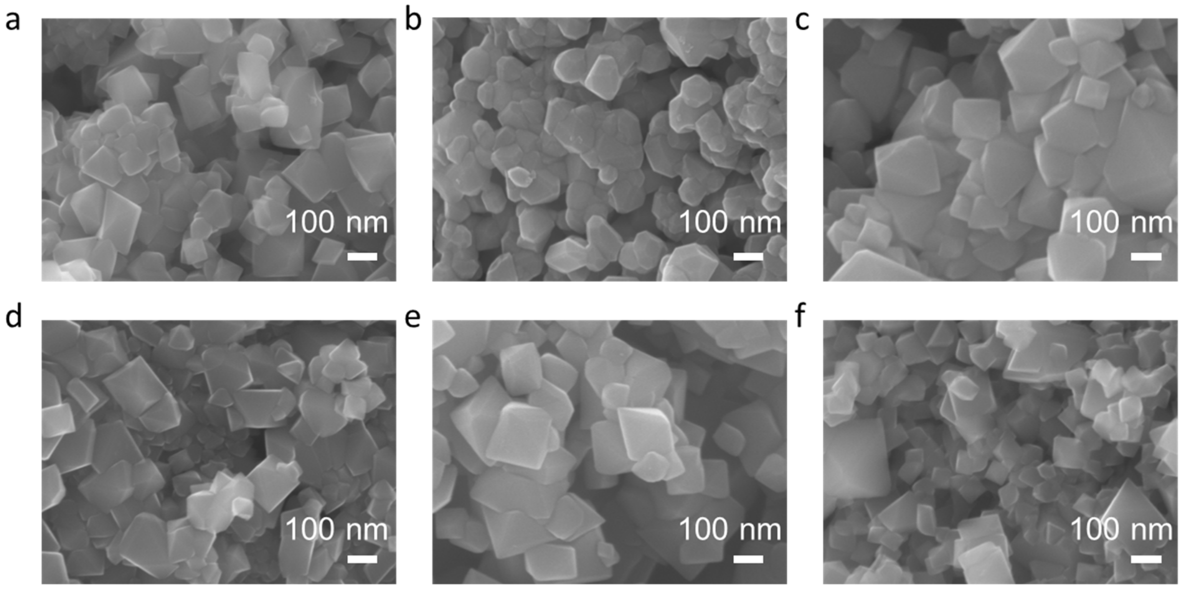

Supplementary Figure 5. Influence of different metals on the LNMO morphology. SEM images of LNMO (a), LNMO-D0.5La (b), LNMO-C0.5La (c), 0.5 at% Al-doped LNMO (LNMO-D0.5Al, d), 0.5 at% Ga-doped LNMO (LNMO-D0.5Ga, d) and 0.5 at% Y-doped LNMO (LNMO-D0.5Y, e), The scale bar corresponds to 100 nm. As can be seen, only La doping conspicuously changes the morphology of the crystals, resulting in smooth edges.

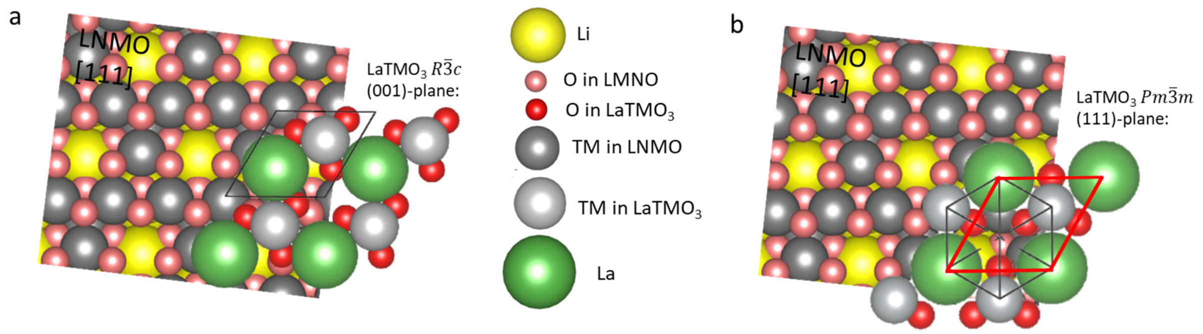

Supplementary Figure 6. Potential epitaxial match of the rhombohedral $R\bar{3}c$ and cubic $Pm\bar{3}m$ structures on-top of the LNMO (111) surface. (a) Projections of the (001) trigonal plane and the cubic (111) plane show arrangements of La atoms in parallelograms matching by about 5% lattice mismatch the hexagonal surface unit cell of LNMO (111). (b) Match even between the oxygen position of the cubic structures. This eventually explains the nucleation of the cubic phase starting with an epitaxial layer when the coating procedure is applied.

Depending on the applied process, the excess of La (typically beyond 0.5 at% La vs TM content) leads either to the formation of a rhombohedral $R\bar{3}c$ (a = 0.551 nm, c = 1.330 nm) $LaTMO_3$ perovskite phase (bulk doping) or to a cubic $Pm\bar{3}m$ (a = 0.388 nm) one (surface coating). The latter structure tends to convert or co-exist with the rhombohedral one for La contents above 1.0 at%. The unit cells of both phases differ only slightly. With c/a=2.414, the $R\bar{3}c$ phase has dimensions close to c/a=$\sqrt{6}$=2.449 where it could match a cubic unit cell of comparable lattice parameter then the observed cubic phase. The split peak at 2.3 $Å^{-1}$ corresponds to the d-spacings of the rhombohedral (2-10) and (104) lattice planes. For c/a=$\sqrt{6}$=2.449 those would become indistinguishable and correspond to the cubic (110) planes. A similar epitaxial match exists for the (001) facet (see Supplementary Figure 7b).

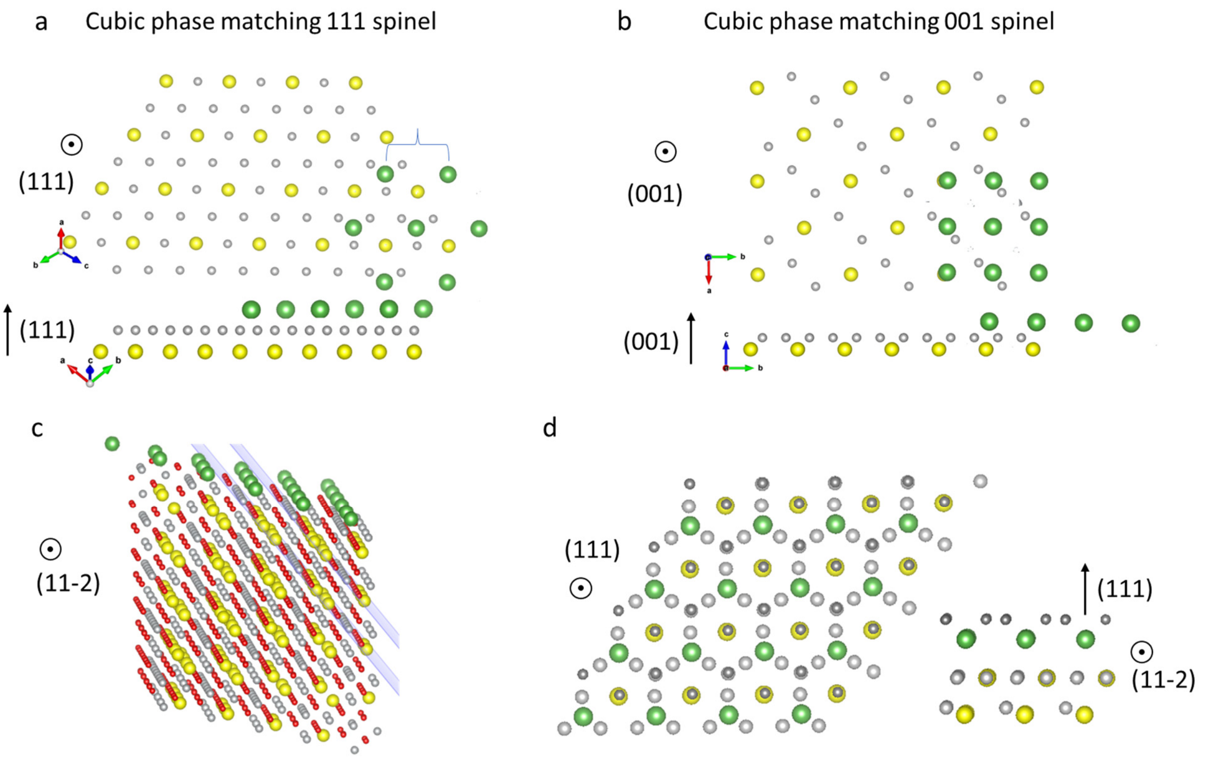

Supplementary Figure 7. Epitaxial match between LNMO and cubic $LaTMO_3$ from different planes. (a) Epitaxial match between the LNMO spinel (111) facet and the La arrangement in the $LaTMO_3$ cubic perovskite phase (plane projection and side view). Note that the oxygen atoms are not shown. (b) Same situation as in (a) for the (001) facet. (c) Representation of La coverage of one (111) facet of an octahedron. (d) Surface structure obtained from DFT modelling for La monolayer deposited as $LaNiO_3$ on $LiMn_2O_4$. The positioning of the La atoms is found to be in perfect epitaxial match (no lattice parameter difference) and comes very close to the suggested match in (a).

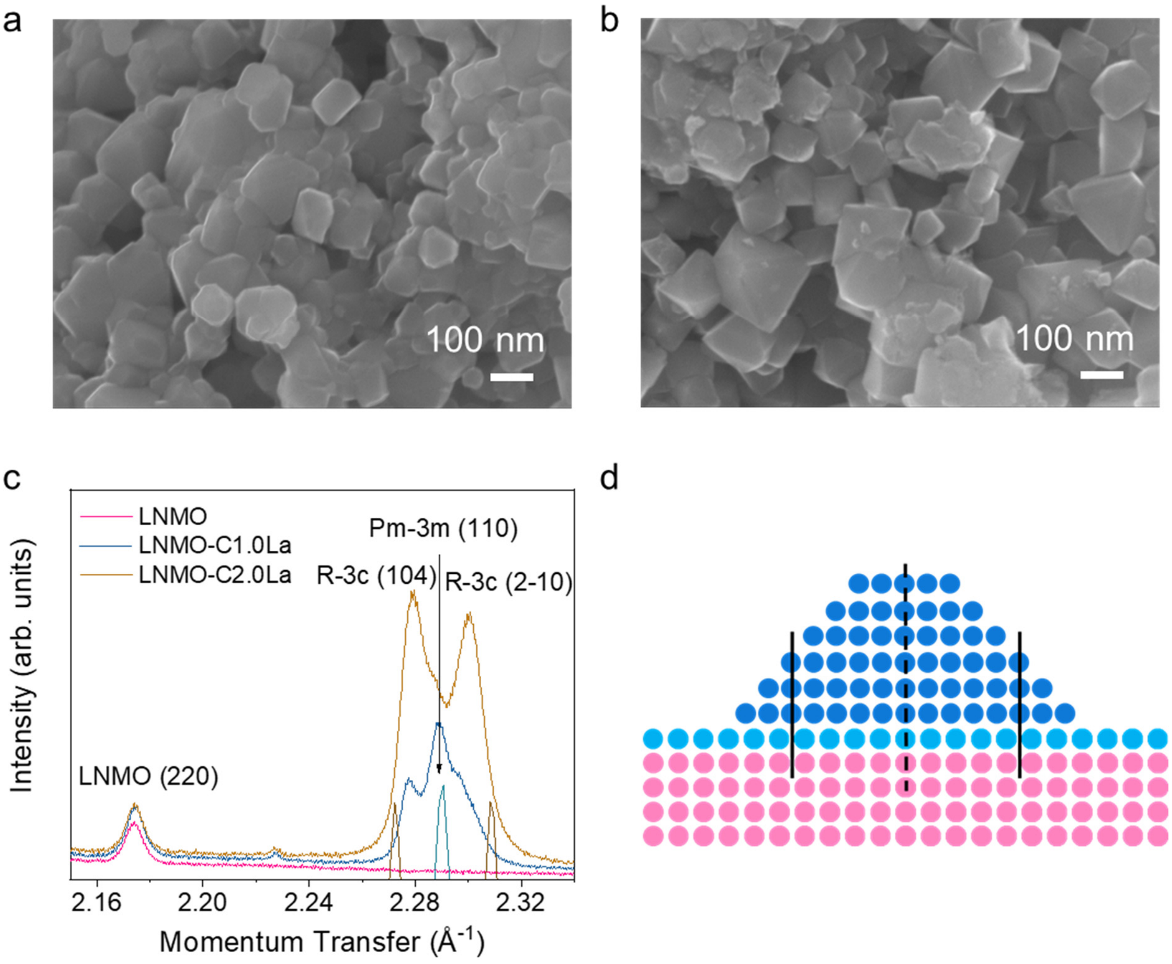

Supplementary Figure 8. Growth model of $LaTMO_3$ on LNMO substrate. (a) SEM image of LNMO-C1.0La. (b) SEM image of LNMO coated 2.0 at% La (LNMO-C2.0La). (c) Zoom on the XRD patterns between the LNMO (220) peak and the $LaTMO_3$ cubic (110) reflection. Up to 1.0 at% La coating, the growth of a cubic $LaTMO_3$ phase dominates. At 2.0 at% La coating, the rhombohedral phase dominates. The theoretical peak positions of these phases are shown in cyan (cubic) and brown (rhombohedral). (d) Schematic illustration of the growth of $LaTMO_3$ on the facets of a LNMO crystal (pink): the first deposited monolayer (light blue) adopts the surface unit cell of the LNMO (111) facet, the following layers (deep blue) grow as fully relaxed islands. The 5 % lattice mismatch to the LNMO lead to a periodic network of interface defects (full vertical lines) and coincidences (dashed vertical lines) typical for semi-coherent interfaces.

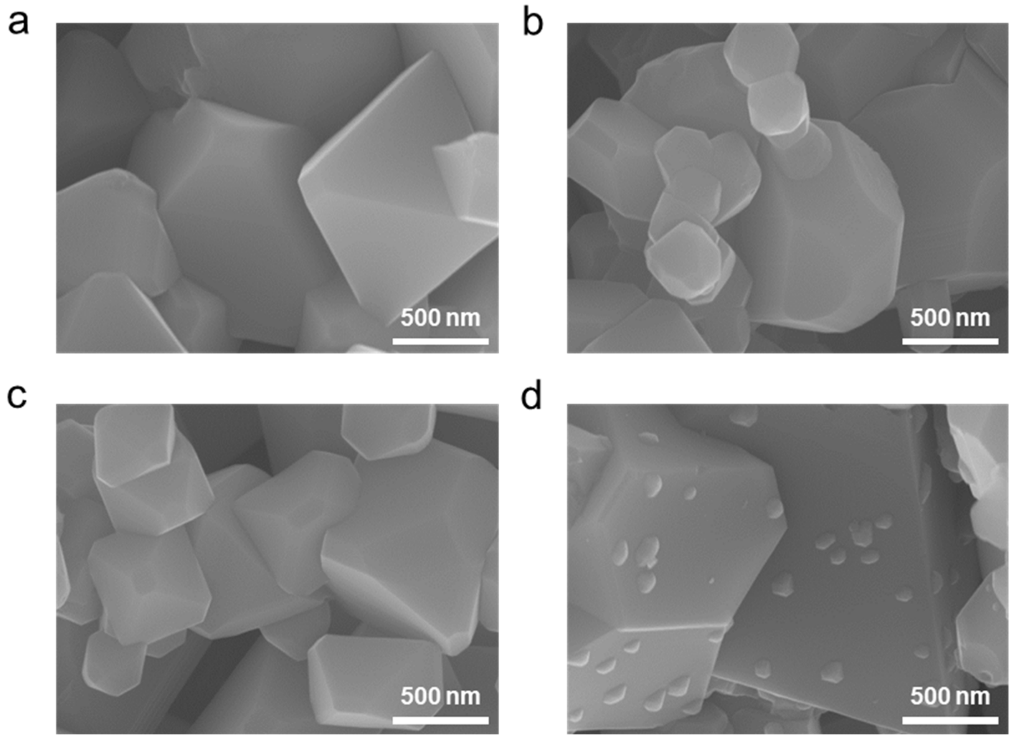

Supplementary Figure 9. The influence on morphology of BLNMO by La integration. SEM images of BLNMO (a), BLNMO-D0.5La (c), BLNMO-C0.5La (c) and BLNMO-C1.0La (d).

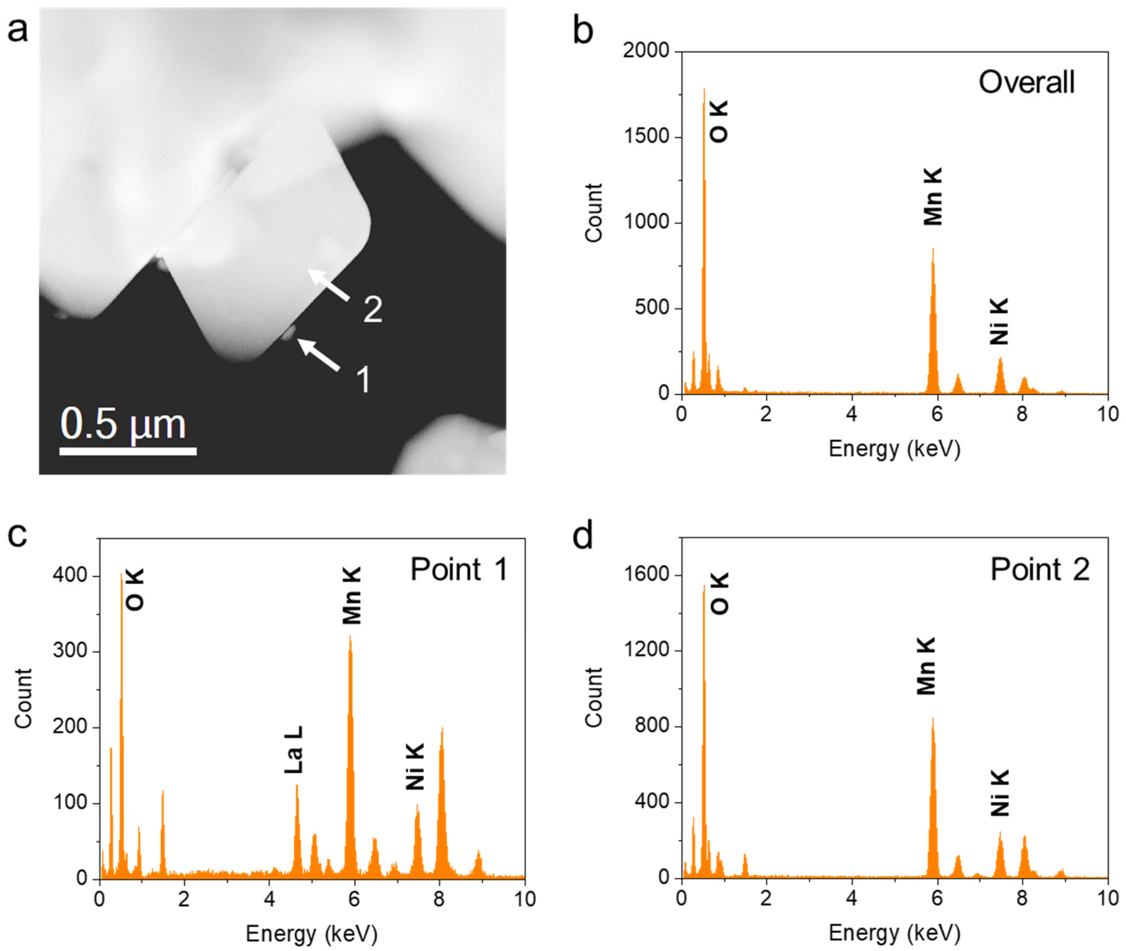

Supplementary Figure 10. EDS analysis of BLNMO-C1.0La from the surface island to the bulk phase. (a) HAADF-STEM image of BLNMO-C1.0La. (c-d) EDS spectra of the overall region (b), point 1 (island, c) and point 2 (bulk, d).

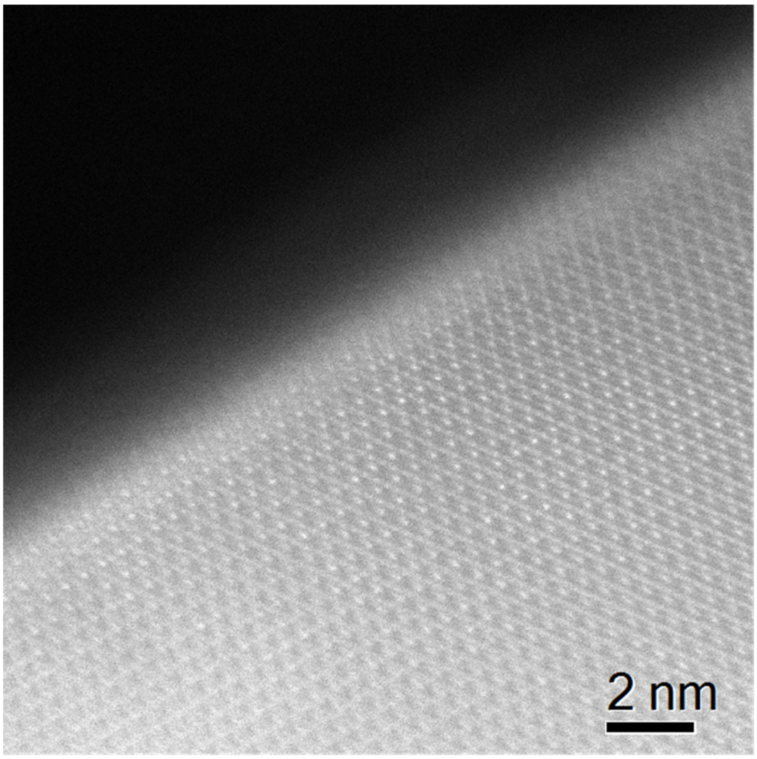

Supplementary Figure 11. HAADF-STEM image of pristine LNMO crystals from [110] axis showing “smooth” (111) edge plane.

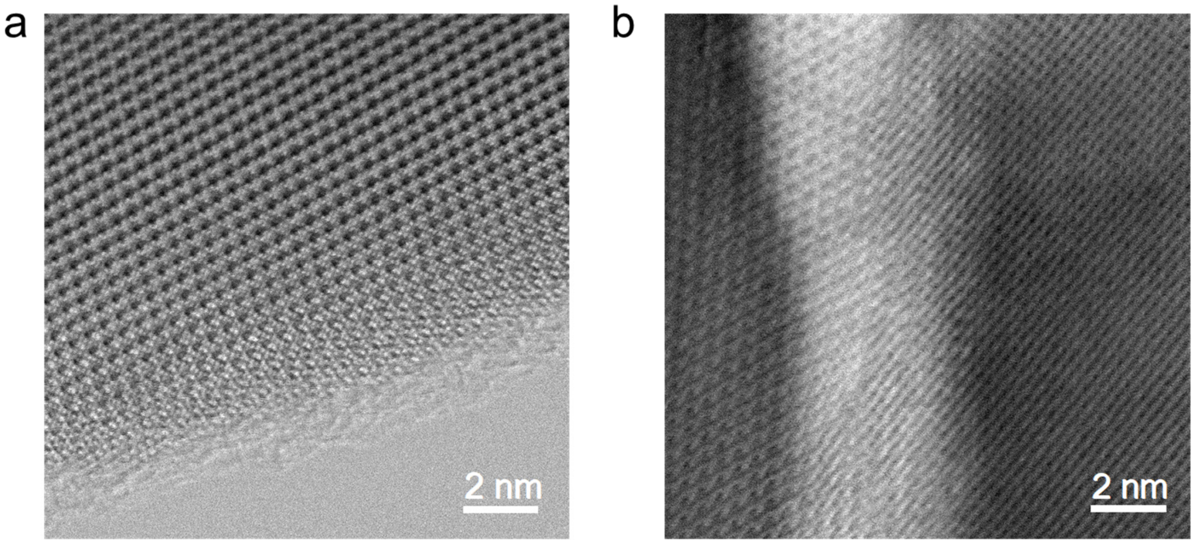

Supplementary Figure 12. (a) ABF-STEM image taken at the edge of LNMO-C0.5La, (b) ABF-STEM image of BLNMO-C1.0La captured at the boundary between the spinel and the La-rich surface island. The discontinued outer layer and the connection between bulk and surface phases are consistent with the HAADF-STEM observations. The islanded secondary phase can be directly imaged by STEM, which evidences the growth mode together with the quantitative X-ray methods.

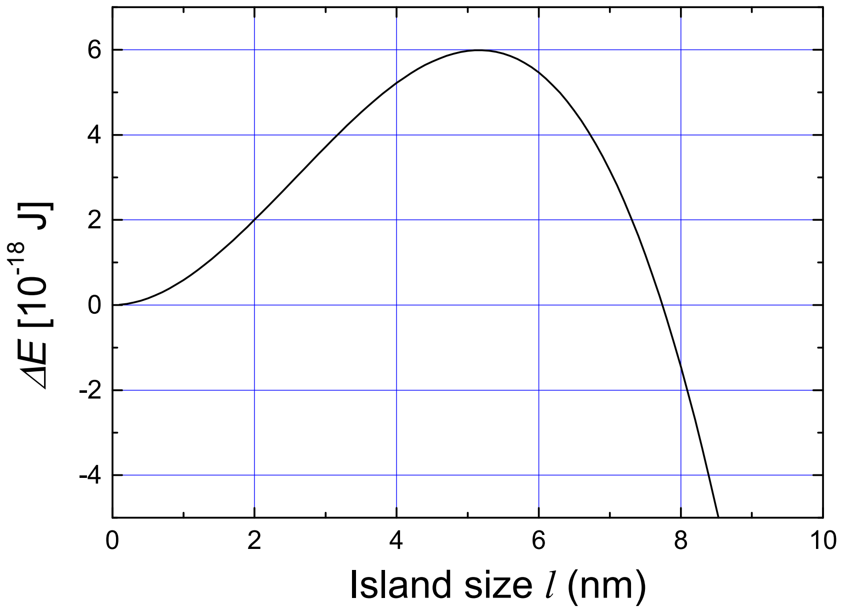

Supplementary Figure 13. Energy consideration for the growth of $LaTMO_3$ islands. Energy difference $\boldsymbol{\Delta E}$ caused by the formation of a trigonal pyramid shaped $LaTMO_3$ island with three {001} facets and base length $\boldsymbol{l}$.

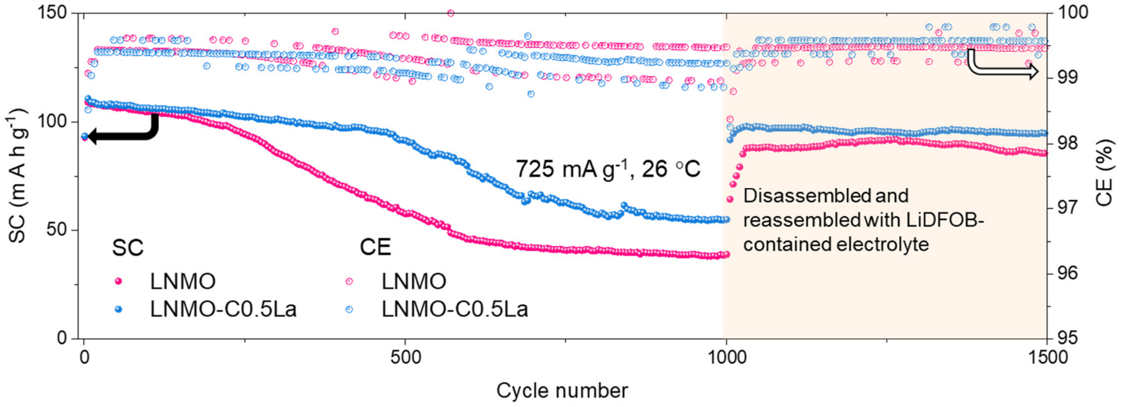

Supplementary Figure 14. Cycling performance of LNMO and LNMO-C0.5La in half cells **at** 26 °C influenced by electrolyte. SC and CE represent specific capacity and Coulombic efficiency. LNMO-C0.5La shows improved cycling stability in the standard electrolyte without LiDFOB The capacity drop of LNMO is significant after 200 cycles while LNMO-C0.5La remains stable before 500 cycles. After 1000 cycles, the capacity retention ratios of LNMO and LNMO-C0.5La are 36.8% and 51.9%, respectively. The cycled cells were disassembled in glove box, and the cathodes were collected and directly reassembled into new cells with the LiDFOB-contained electrolyte. Notably, the capacity of the electrodes can be recovered and remain stable for another 500 cycles, indicating the capacity drop is due to the decomposition of the standard electrolyte.

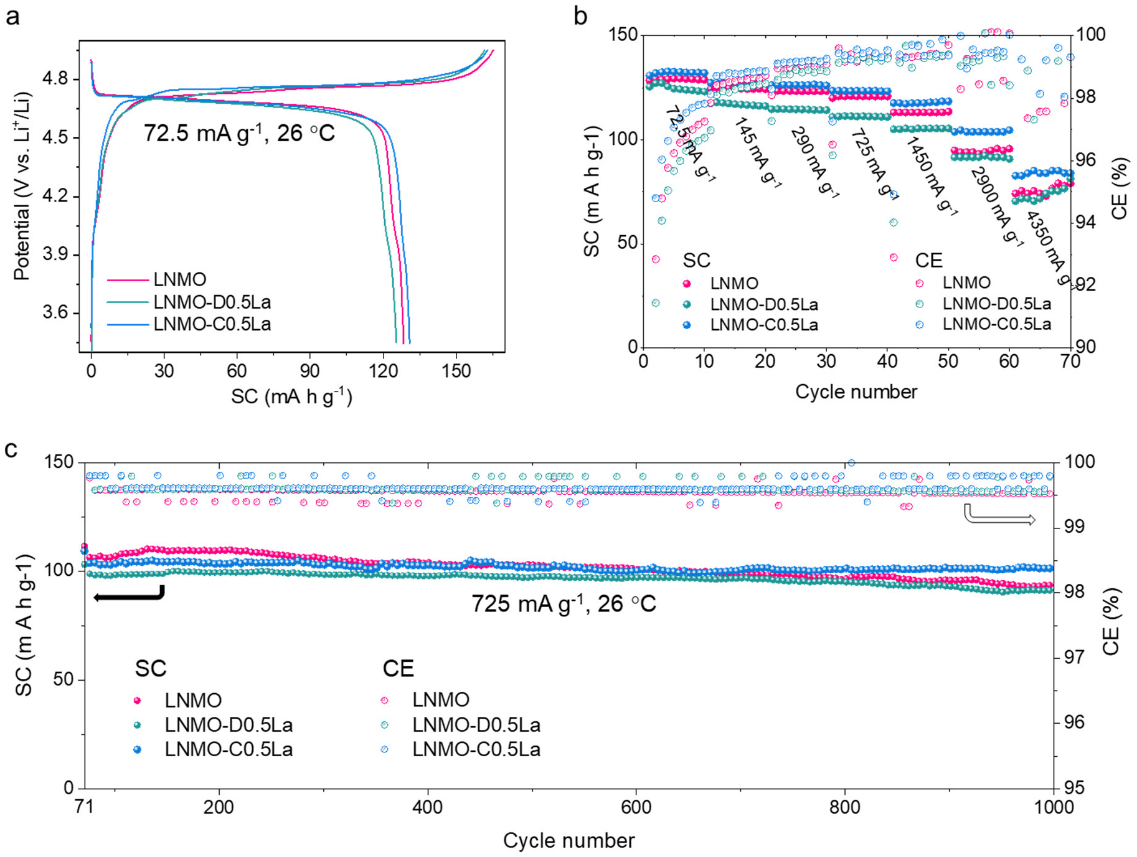

Supplementary Figure 15. **Electrochemical performance of LNMO, LNMO-D0.5La and LNMO-C0.5La in half-cell configuration at 26 °C.** (a) Initial charge/discharge profiles of the LNMO cathodes at 72.5 mA g^{-1}. (b) Discharge capacities of the LNMO cathodes at different specific current (the charge specific current is fixed at 145 mA g^{-1} except the discharge specific current is 72.5 mA g^{-1}). (c) Long cycling performance of Li||LNMOs half cells at a charging/discharging specific current of 725 mA g^{-1}

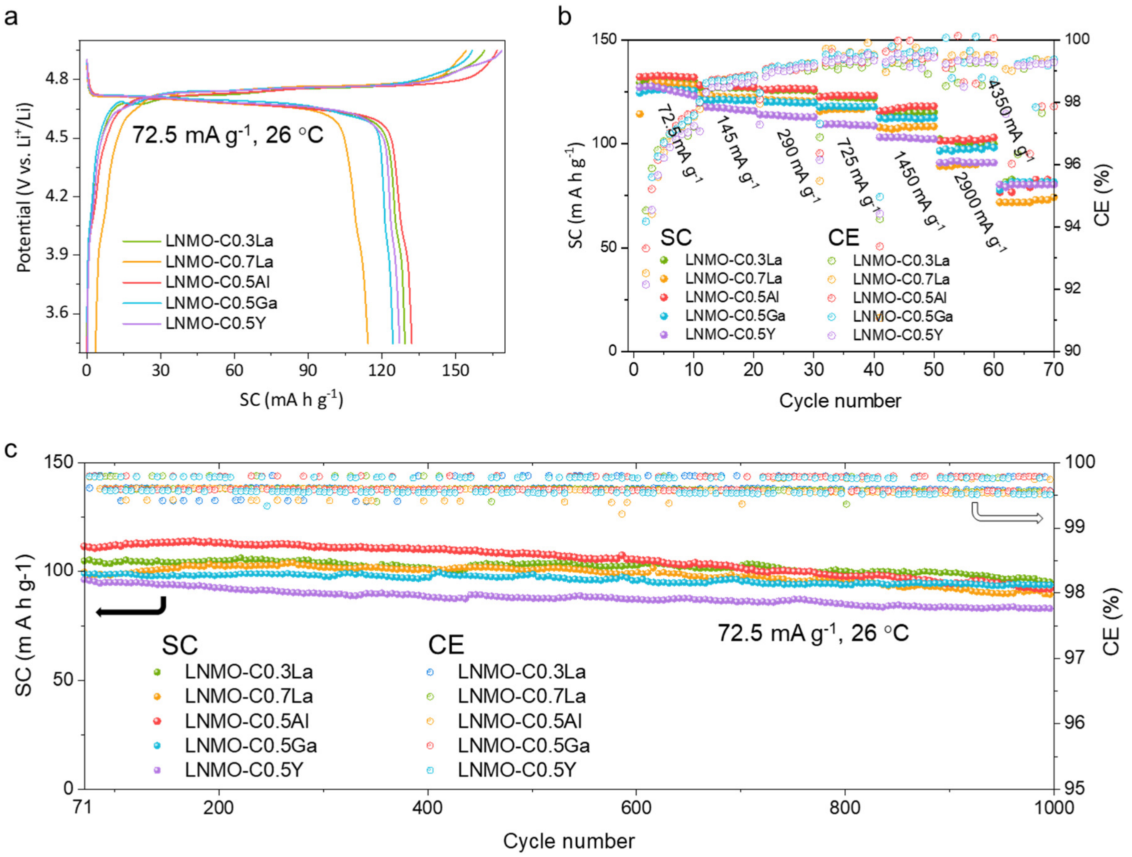

Supplementary Figure 16. Electrochemical performance of LNMOs coated with different metals in half-cell configuration at 26 °C. (A) Initial charge/discharge profiles of the LNMO cathodes at a charging/discharging specific current of 72.5 mA g^{-1}. (B) Capacities of the LNMO cathodes at different specific currents. (C) Long cycling performance of Li||LNMOs cells at 725 mA g^{-1}.

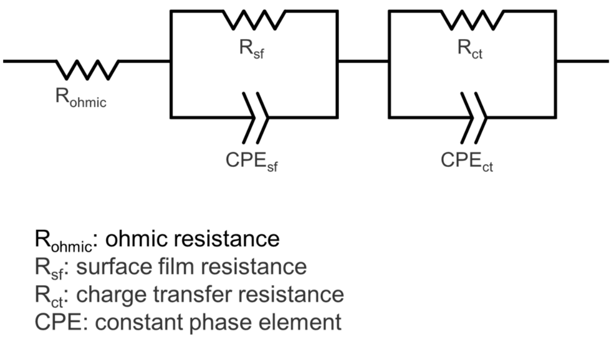

Supplementary Figure 17. Equivalent circuit model for EIS data fitting (*6*).

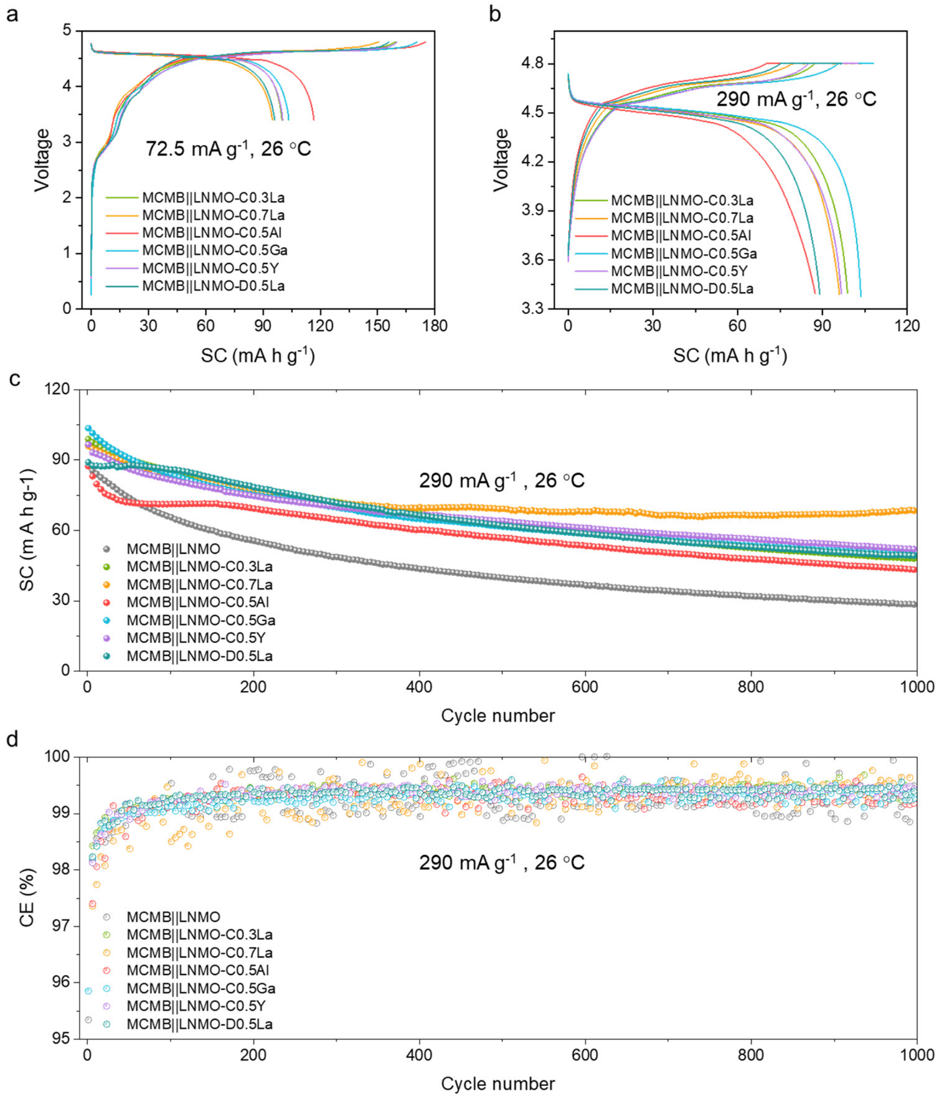

Supplementary Figure 18. Electrochemical performance of LNMOs in full cells with MCMB graphite as the anode at 26 °C. (a) Initial charge/discharge profiles of the full cells with different LNMOs. (b) The charge/discharge profiles of the full cells at 290 mA g^{-1} (following constant current charge at 290 mA g^{-1}, an additional constant 4.8 V charge is applied until the current drops to 29 mA g^{-1}). (c) Long cycling performance of MCMB||LNMOs cells at 290 mA g^{-1} (the cells were pre-cycled at 72.5 mA g^{-1} for 5 cycles). LNMO-C0.5Al, LNMO-C0.5Ga and LNMO-C0.5Y represent LNMO coated by 0.5 at% Al, Ga and Y, respectively. (d) CE of the cells over the 1000 cycles.

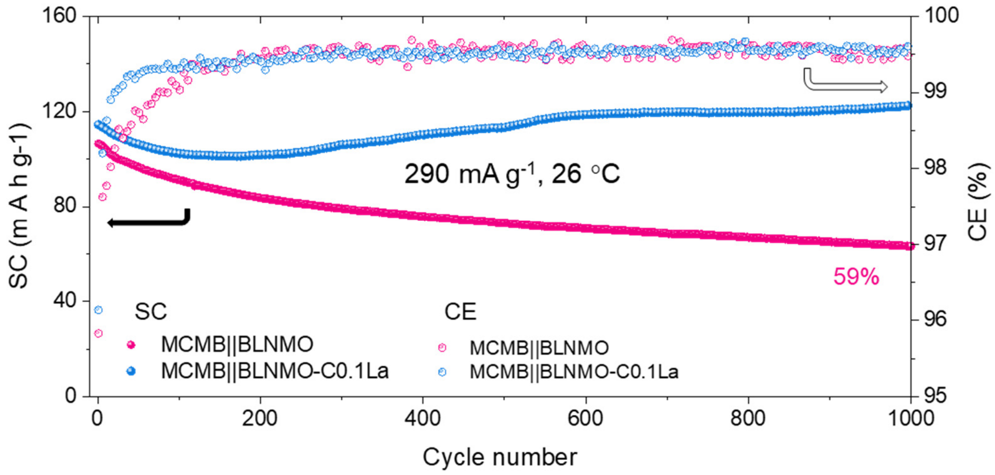

Supplementary Figure 19. Cycling performance of BLNMOs in full cells at 26°C. Cycling performance of BLNMO and BLNMO-C0.1La in full cells using MCMB graphite as the anode (the cells were pre-cycled at 72.5 mA g^{-1} for 5 cycles).

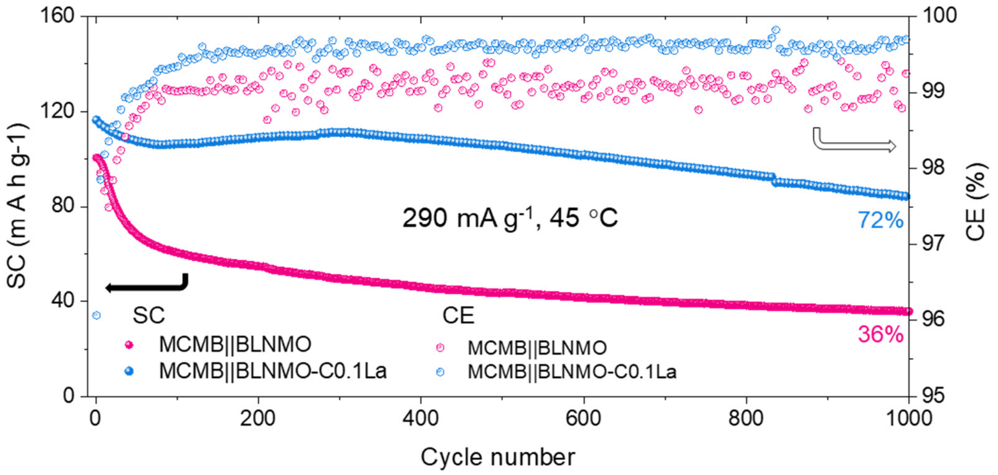

Supplementary Figure 20. Cycling performance of the BLNMO and BLNMO-C0.1La in full cells at 45 °C (the cells were pre-cycled at 72.5 mA g^{-1} for 5 cycles).

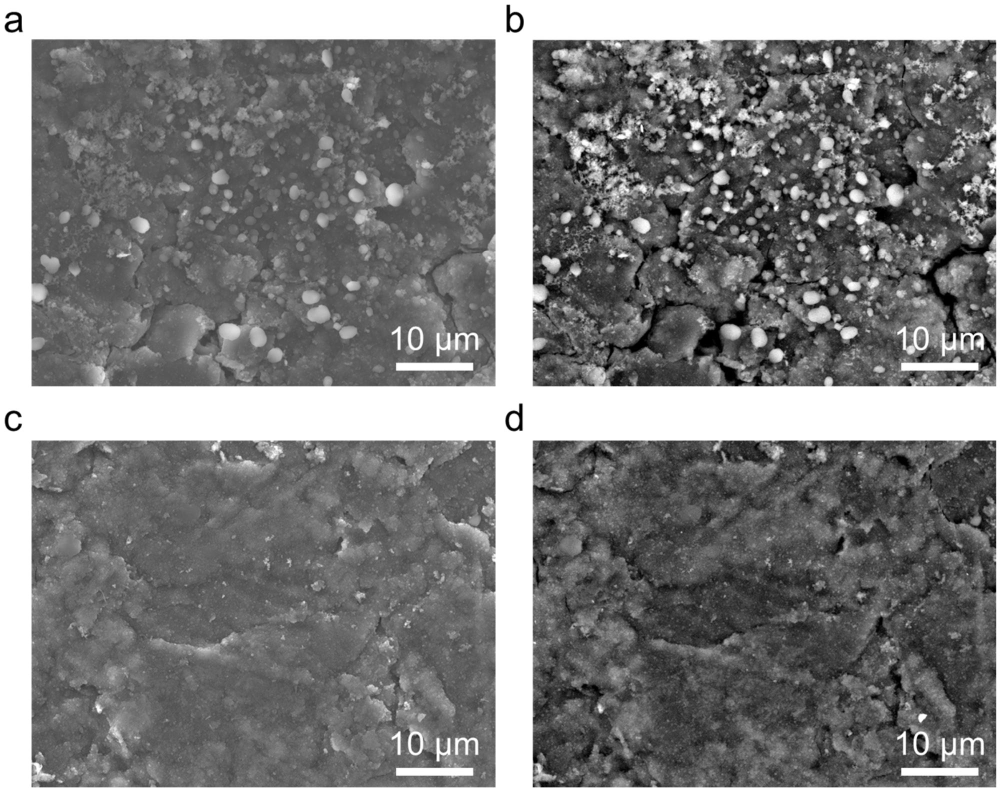

Supplementary Figure 21. (a, b) SEM images of cycled graphite anode worked with LNMO cathode detecting secondary electrons (a) and backscattered electrons (b). (c, d) SEM images of cycled graphite anode worked with LNMO-C0.5La cathode detecting secondary electrons (c) and backscattered electrons (d). The anodes were collected from full cells at discharged state after 1000 cycles at 290 mA g^{-1} under 26 °C.

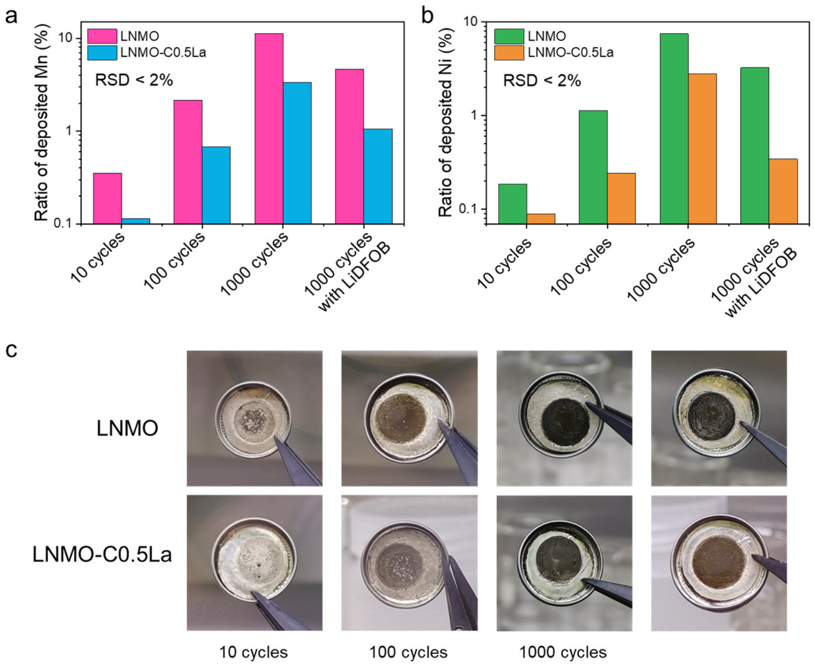

Supplementary Figure 22. Suppressed TM dissolution enabled by cathode surface passivation. (a, b) Calculated ratios of deposited Mn (a) and Ni (b) with respect to the Mn and Ni in the cathode materials by harvesting the Li metal anodes at charged state. The relative standard deviation (RSD) of the measurement was less than 2%. The cells were cycled 1000 times at 725 mA g^{-1} under 26 °C. (c) Corresponding digital photographs of the Li electrodes.

At charge state, the dissolved Mn and Ni are expected to be deposited on the Li foil:
Cathode:

$$LiNi_{0.5}Mn_{1.5}O_4 = Ni_{0.5}Mn_{1.5}O_4 + Li^+ + e^-$$

Anode:

$$Ni^{2+} + 2e^- = Ni \quad E = ca. 2.79\ V\ vs\ Li^+/Li$$

$$Mn^{2+} + 2e^- = Mn \quad E = ca. 1.855\ V\ vs\ Li^+/Li$$

$$Li^+ + e^- = Li \quad E = 0\ V\ vs\ Li^+/Li$$

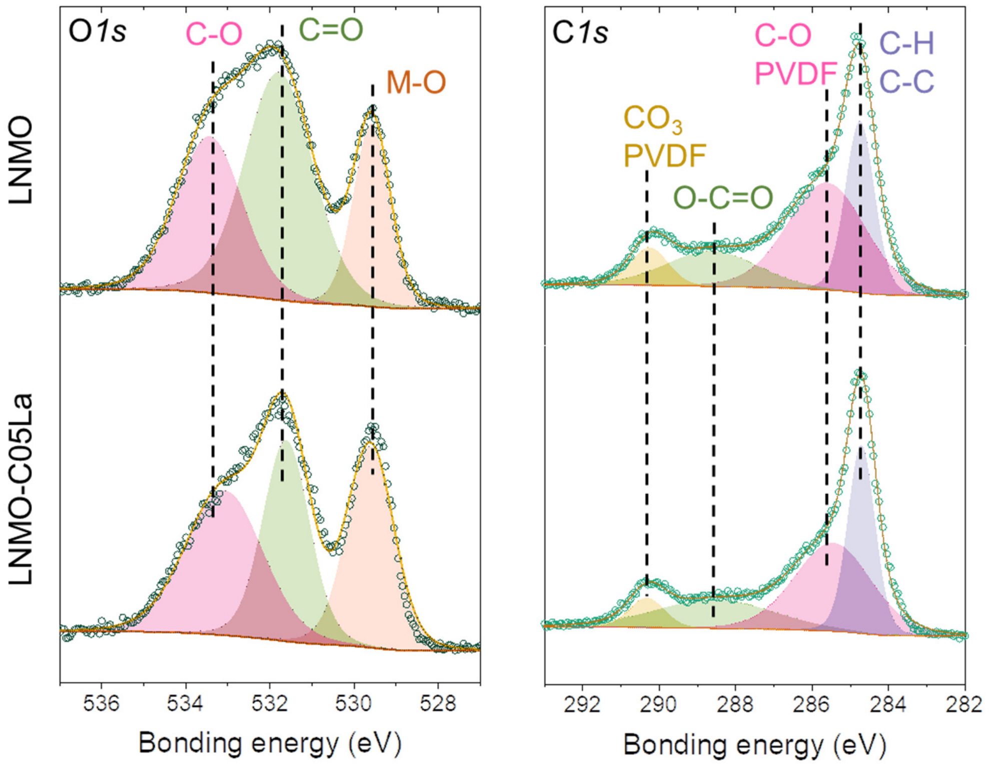

Supplementary Figure 23. Evolution of the cathode surface chemistry after cell cycling. XPS spectra (O*1s* and C*1s*) obtained from the cathodes collected from MCMB||LNMO (top) and MCMB||LNMO-C0.5La (bottom) full cells. In the O*1s* spectra, the intensity of lattice M-O is much stronger for cycled LNMO-C0.5La cathode, suggesting the reduced cathode-electrolyte reactions owing to $LaTMO_3$ surface passivation. All the electrodes were collected from full cells at discharged state after 100 cycles at 290 mA g^{-1} under 26 °C.

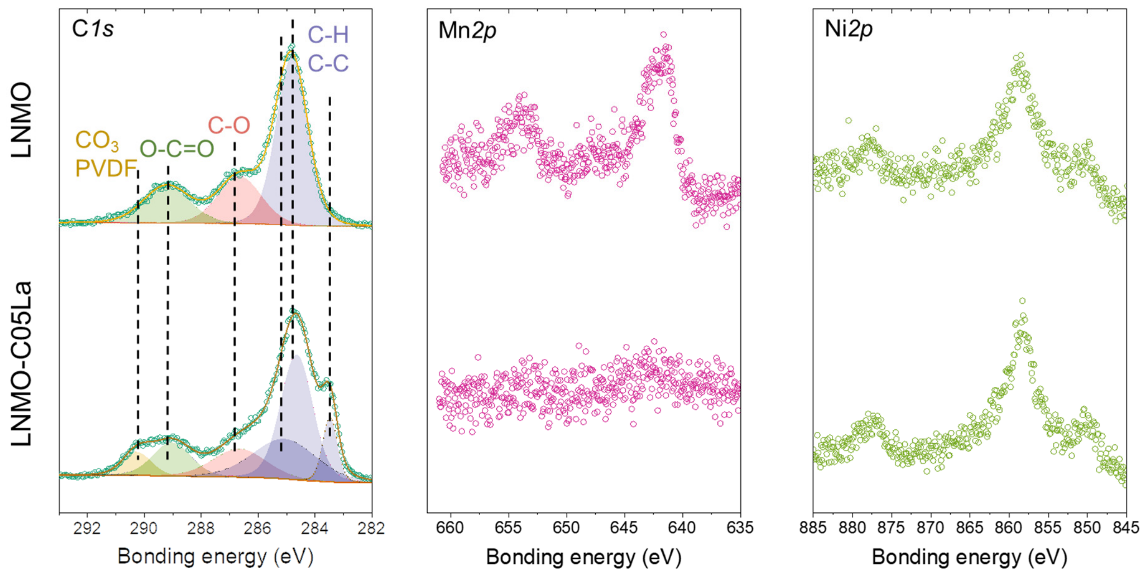

Supplementary Figure 24. Evolution of the anode surface chemistry after cell cycling. XPS spectra (C*1s*, Mn*2p* and Ni*2p*) obtained from the graphite anodes collected from MCMB||LNMO (top) and MCMB||LNMO-C0.5La (bottom) full cells after 100 cycles at 290 mA g^{-1}. The C*1s* spectra for cycled graphite anode involve C-C/C-H from graphite, F-C-F from PVDF and C-O, O-C=O, O–CO_2 from the typical solid electrolyte interphase (SEI) components, such as ROLi, RCOOLi and $ROCO_2Li$ *(7)*. Clearly, the graphite anode paired with LNMO-C0.5La shows weaker SEI featured peaks, suggesting less SEI formation. Furthermore, the signal of Mn*2p* from the anode part is hardly detectable when the cathode is LNMO-C0.5La. Ni dispostion on the anode is similar for both cells at discharged state.

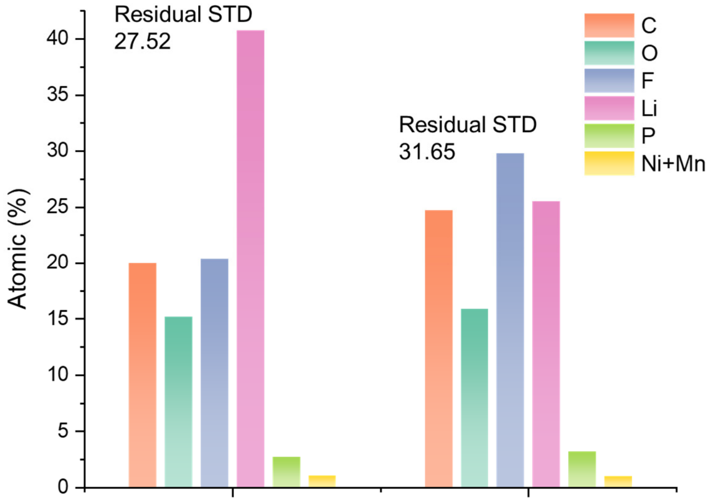

Supplementary Figure 25. The role of cathode passivation on changing the anode surface composition. The relative atomic ratios of different elements on the surface of graphite anodes in MCMB||LNMO (left) and MCMB||LNMO-C0.5La (right) full cells after 100 cycles at 290 mA g^{-1}. The residual standard deviations (STDs) are 27.52 and 31.65, respectively. The most evident feature is that the atomic ratio of Li is much lower for the MCMB||LNMO-C0.5La full cell, which indicates that the Li trapping effect is alleviated when the cathode is passivated by $LaTMO_3$.

Supplementary Table 1 Quantitative results from the EDS analysis.

	Ni K at%	Mn K at%	La L at%	O K at%	C K at%
Point 1 (island)	5.03	18.58	5.70	33.56	37.13
Point 2 (bulk)	5.08	17.45	0	50.63	26.84
Overall	4.82	19.68	0.17	57.38	17.95

Supplementary Table 2 Key results obtained from EIS fitting.

	MCMB||LNMO after 1 cycle	MCMB||LNMO-C0.5La after 1 cycle	MCMB||LNMO after 1000 cycles	MCMB||LNMO-C0.5La after 1000 cycles
R_{ohmic} (Ω)	9.94	7.28	6.84	5.51
R_{ohmic} error (%)	4.28	2.24	5.05	2.35
R_{sf} (Ω)	23.4	16.8	120.0	38.7
R_{sf} error (%)	4.39	2.59	0.67	2.10
R_{ct} (Ω)	62.7	38.0	146.0	74.4
R_{ct} error (%)	3.92	3.17	3.39	2.43
Overall error (%)	2.54	1.78	1.90	2.22

Supplementary References

1. B. Voigtländer, Fundamental processes in Si/Si and Ge/Si epitaxy studied by scanning tunneling microscopy during growth. *Surface Science Reports* **43**, 127-254 (2001).
2. T. U. Schülli, M. Sztucki, V. Chamard, T. H. Metzger, D. Schuh, Anomalous x-ray diffraction on InAs/GaAs quantum dot systems. *Applied Physics Letters* **81**, 448-450 (2002).
3. T. U. Schülli, J. Stangl, Z. Zhong, R. T. Lechner, M. Sztucki, T. H. Metzger and G. Bauer, Direct Determination of Strain and Composition Profiles in SiGe Islands by Anomalous X-Ray Diffraction at High Momentum Transfer. *Physical Review Letters* **90**, 066105 (2003).
4. D. N. Lee, Elastic properties of thin films of cubic system, *Thin Solid Films* **434**, 183-189 (2003).
5. R. Gaillac, P. Pullumbi, F.-X. Coudert, ELATE: an open-source online application for analysis and visualization of elastic tensors. *Journal of Physics: Condensed Matter* **28**, 275201 (2016) & http://progs.coudert.name/elate/mp?query=mp-19025.
6. An SJ*, et al.* Electrolyte Volume Effects on Electrochemical Performance and Solid Electrolyte Interphase in Si-Graphite/NMC Lithium-Ion Pouch Cells. *ACS Applied Materials & Interfaces* **9**, 18799-18808 (2017).
7. S. J. An, J. Li, C. Daniel, D. Mohanty, S. Nagpure and D. L. Wood, The state of understanding of the lithium-ion-battery graphite solid electrolyte interphase (SEI) and its relationship to formation cycling. *Carbon* **105**, 52-76 (2016).
